# Airfreight data from Memanbetsu airport correlated to fish and scallop catch in okhotsk subprefecture, Hokkaido, Japan

**DOI:** 10.1016/j.dib.2020.106006

**Published:** 2020-07-07

**Authors:** Hiroshi Matsumura, Tomohisa Ueda, Yoshimasa Sagane

**Affiliations:** a Department of Business, Natural Resource and Economic Development, Tokyo University of Agriculture, 196 Yasaka, Abashiri, Hokkaido 099-2493, Japan; b Department of Food, Aroma and Cosmetic Chemistry, Tokyo University of Agriculture, 196 Yasaka, Abashiri, Hokkaido 099-2493, Japan

**Keywords:** Airport, Logistics, Fishing industry, Supply chain, Regional industry

## Abstract

This data article features four figures showing the correlations between the biomass of fish and scallop landed in Okhotsk subprefecture (Hokkaido, Japan) and airfreights from Memanbetsu Airport, a dominant airport in the subprefecture. Total landings of fish and scallop were collected from Hokkaido government office website while the airfreight data were acquired from the Memanbetsu Airport management office. The second and third figures in this data article are in a matrix heatmap format and a line graph, representing the statistical monthly data of airfreights from the airport and fish and scallop landings in the subprefecture; data were gathered from 2009 to 2018. Data were gathered from 2009 to 2018. The airfreight data reflect the increase and decrease in the total fish or scallop landings in the subprefecture. A Pearson's correlation coefficient and cross-correlation statistics between monthly airfreight and monthly fish landings were performed to assess their correlation and annual periodic relationship. This article also provides a data figure that shows an increasing active opening ratio to secure human resources in the motor truck transportation industry in Japan. This may be due to a workforce shortage in this trade. Annual data on the active opening ratio for jobs in Japan from 2001 to 2018 were collected from the open data available on the website of the Japanese Ministry of Health, Labour and Welfare.

Specifications table

**Table:** 

Subject	Social Sciences
Specific subject area	Logistics
Type of data	Table and Graph
How data were acquired	Raw statistical data were acquired from the Memanbetsu Airport management office and the Hokkaido government office's website. Analyzed data were computed through statistical software: Microsoft Excel and R.
Data format	Raw and Analyzed
Parameters for data collection	Monthly data of both domestic airfreight from Memanbetsu Airport and monthly data of total fish and scallop landings at Okhotsk Subprefecture, Hokkaido, Japan were collected from January 2009 to December 2018; data from each time period were compared
Description of data collection	Airfreight data was kindly provided by the Memanbetsu Airport Office. Data of total fish landings and scallop landings were collected from the Hokkaido Prefecture Government website. Correlation between the amount of airfreight in Memanbetsu airport and fish or scallop landings were computed by correlation analysis using statistical software. The annual active opening ratio data for jobs in Japan were collected from the open data on the Ministry of Health, Labour and Welfare's website.
Data source location	City/Town/Region: Okhotsk Subprefecture, Hokkaido, Japan.
Data accessibility	With the article

## Value of the data

The data exhibiting the tight correlation between airfreight logistics and total fish landing in the local area could improve understanding of the current status of the logistics of agricultural and marine products.
The data represented could benefit researchers in rural economics, agricultural logistics, and agricultural policy.
In Japan, traffic network in this agricultural and fishery area is decreasing due to termination in some sections of railway network and serious shortage of truck drivers due to the aging population and challenging working environment, despite the importance of agricultural logistics for the whole country. The data can be valuable for planning agricultural logistics in Japan, given the imbalanced relationship between logistics and agricultural and marine production.

## 1 Data description

**Fig. 1:**
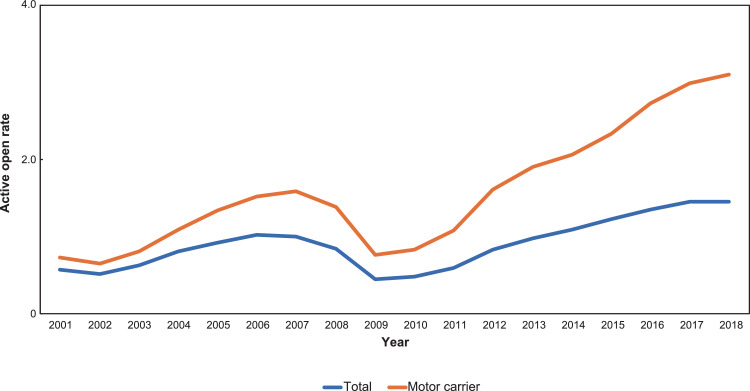
Line graph representing the annual data of active opening ratio for jobs in Japan. The ratios for jobs in the motor truck transportation industry (orange) and for all industries (blue) are shown.

**Fig. 2:**
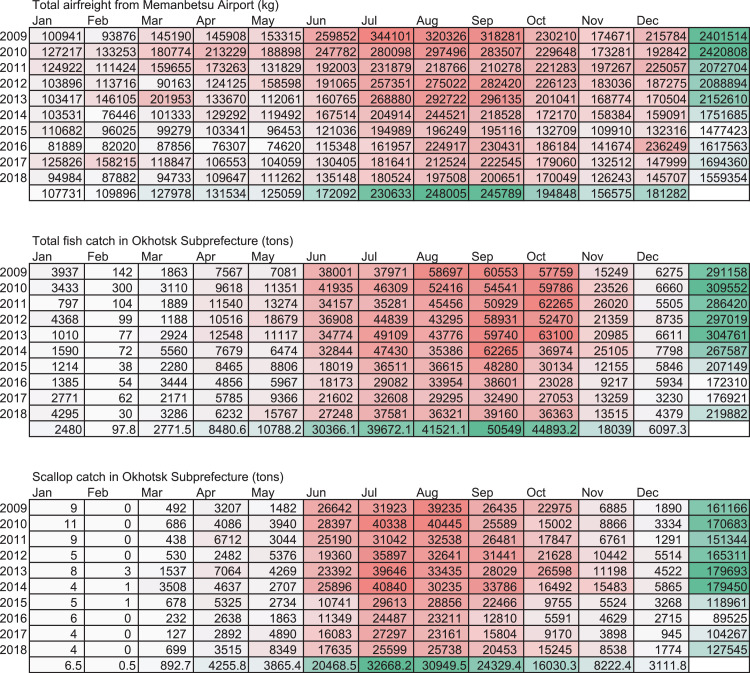
Heatmap representing the total airfreight (kg) from Memanbetsu Airport and the total fish (tons) and scallop (tons) landings in Okhotsk subprefecture. Raw data for this figure are also provided as supplementary material to this article.

**Fig. 3:**
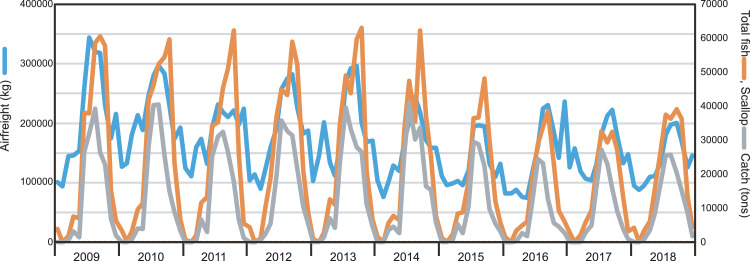
Line graph representing the monthly data on total airfreight (kg) from Memanbetsu Airport and the total fish (tons) and scallop (tons) landings in Okhotsk subprefecture.

**Fig. 4:**
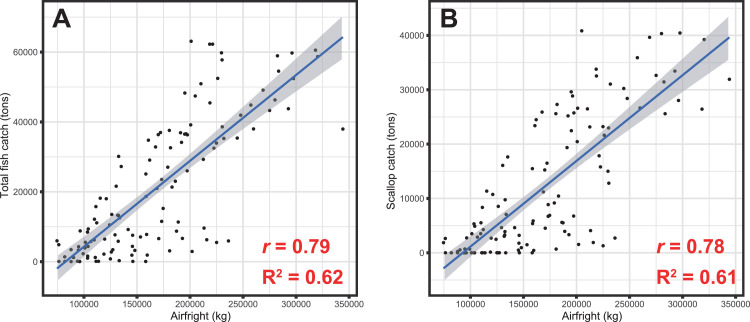
Scatter plot showing correlations between the total airfreight from Memanbetsu Airport and the total fish catch (A) and scallop catch (B). Linear regression (blue line) is shown with 95% confidence intervals (gray area). The coefficient of determination (*R^2^*) and the Pearson's correlation coefficient (*r*) with *p*-value < 0.05 are indicated in each panel.

**Fig. 5:**
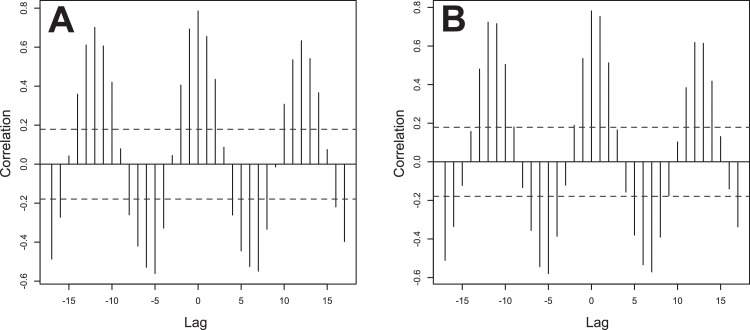
Cross-correlation analysis between total airfreight from Memanbetsu Airport and total fish (A) or scallop (B) landings. Vertical lines (black line) indicating the Pearson's correlation coefficient with *p*-value < 0.05 in each time-lag are shown with 95% confidence intervals (dotted lines).

This data article provides five sets of data in figures. Fig. 1 is a line graph showing the annual active opening ratio (ratio of job openings to job applicants) in Japan. In the graph, blue line indicates the ratio for total jobs on average, while the orange line indicates the ratio for jobs in the motor truck transportation industry, from 2001 to 2018. Fig. 2 shows three matrix heatmaps. The first map shows data of the total monthly airfreight quantities transported from Memanbetsu Airport (Hokkaido, Japan) to New-Chitose Airport (Hokkaido, Japan), Okadama Airport (Hokkaido, Japan), Tokyo International Airport (Tokyo, Japan), Chubu Centair International Airport (Aichi, Japan), Osaka International Airport (Osaka, Japan), and Kansai International Airport (Osaka, Japan). The second and third panels show the total fish and scallop landings respectively. The vertical axis indicates the year—2009 to 2018—while the horizontal axis indicates months, from January to December. Fig. 3 is a merged line graph showing the monthly data of total airfreight quantities and total fish and scallop landings. Scatter plots and regression statistics indicate a strong positive linear relationship between airfreight and total fish catch with a coefficient of determination (*R^2^*) of 0.62 and a Pearson's correlation coefficient (*r*) of 0.79, with a *p*-value < 0.05 (Fig. 4A) and between airfreight and scallop catch with an *R^2^* of 0.62 and *r* of 0.79, with a *p*-value < 0.05 (Fig. 4B). Correlations between the time-lagged series (−17 to +17) of airfreight and fish catch indicate the annual periodic relationship of the data (Fig. 5).

## 2 Experimental design, materials, and methods

### 2.1 Design

Hokkaido, an island and the largest prefecture in Japan, plays an important role in Japan's food supply. The proportion of food production (in calories) in Hokkaido compared with the entirety of Japan has been estimated at 20% [1]. Okhotsk subprefecture in Hokkaido is a key area, due to its high fish and agricultural production. The area under cultivation in the Okhotsk subprefecture accounts for 14.5% of the total cultivated area in Hokkaido [2]. Additionally, fish production in the Okhotsk region accounts for 20% of the total production in Hokkaido [2]. Therefore, large quantities of agricultural and fish products are transported from Okhotsk subprefecture to major cities in Japan. Hokkaido is, however, geographically separated from Honshu island (the mainland of Japan) by sea and accessibility between Hokkaido and Honshu is limited to either sea routes or the *Seikan* Tunnel, an undersea railway tunnel that connects the two islands. Okhotsk subprefecture faces the Sea of Okhotsk; the ports in this area are, therefore, not available for agricultural and fish product transportation. In addition, discussions on terminating some sections of the rail network in Hokkaido is underway as these areas are causing a financial loss [3]. Furthermore, as indicated by Fig. 1 that shows increasing active opening ratio to secure the human resources in motor truck transport industry, there is a serious shortage of truck drivers due to the aging population and challenging working environment, resulting in increasing truck fares [3]. The substitution option for transporting these products would be airfreight with the introduction of a low-cost carrier. In our preliminary interview survey with stakeholders, it was stated that airfreight is used only for seafood, especially for scallops, for transport to large cities. In this article, we compared the monthly airfreight from Memanbetsu Airport, the largest airport in Okhotsk region, to total fish and scallop landings in the same area.

### 2.2 Source of data

The monthly data (January 2009–December 2018) for airfreight from Memanbetsu Airport to New-Chitose Airport (Hokkaido, Japan), Okadama Airport (Hokkaido), Tokyo International Airport (Tokyo, Japan), Chubu Centair International Airport (Aichi, Japan), Osaka International Airport (Osaka, Japan), and Kansai International Airport (Osaka, Japan) were provided by the Memanbetsu Airport management office. The monthly data for total fish and scallop landings were collected from the open data available on the Hokkaido Prefecture Government website [4]. The open data are published in Excel format and contain the quantities of annual landings for 63 seafood items (e.g., several types of fish, species of squid, crustaceans, and shellfish species). The landings are described in wet weight when the items were caught, except for seaweeds, which are described in dried weight. The total quantity of fish catches in this study is the sum of the landings of all 63 items. The annual data on the active opening ratio (ratio of job openings to job applicants) in Japan from 2001 to 2018 were collected from the open data on website of Ministry of Health, Labour and Welfare, Japan [5]. The data are published in Excel format and provide monthly data of the active opening ratios in 71 job categories, including the motor truck transportation industry. The ratios were calculated as job openings divided by job applicants.

### 2.3 Heatmap preparing

The number of airfreights and fish catches were obtained from the sources stated above and entered into a Microsoft Excel for Mac worksheet (version 16.33; Redmond, WA, USA), as shown in Fig. 2. The cell colors were determined by the color scale option in Excel, in which the minimum number is indicated in white and the maximum number is indicated in red. The sum of each year and month was calculated using the auto sum option in Excel. The color scale option was separately applied to a line or row of cells for the sum. The minimum number is indicated in white and the maximum number is indicated in green.

### 2.4 Statistical analysis

All statistical analyses were performed using the free statistical software R (available at https://www.r-project.org) [6]. The correlations between airfreight and fish catches were assessed by the scatter plots and regression statistics using the coefficient of determination and Pearson's correlation coefficient (Fig. 4). The scatter plot was created using the *ggplot2* package [7] in R. To detect possible lag on the correlation between airfreight and fish catch quantities, a cross-correlation analysis with time-lagged series (−17 to +17) using the *ccf* command on R was performed (Fig. 5).

## Declaration of Competing Interest

The authors declare that they have no known competing financial interests or personal relationships which have, or could be perceived to have, influenced the work reported in this article.

## Appendix Supplementary materials

Image, application 1

Supplementary material Image, application 2
